# Translational issues for mitoprotective agents as adjunct to reperfusion therapy in patients with ST‐segment elevation myocardial infarction

**DOI:** 10.1111/jcmm.14953

**Published:** 2020-01-22

**Authors:** Hans Erik Bøtker, Hector Alejandro Cabrera‐Fuentes, Marisol Ruiz‐Meana, Gerd Heusch, Michel Ovize

**Affiliations:** ^1^ Department of Cardiology Aarhus University Hospital Aarhus N Denmark; ^2^ SingHealth Duke‐NUS Cardiovascular Sciences Academic Clinical Programme and Cardiovascular and Metabolic Disorders Program Duke‐National University of Singapore Medical School Singapore Singapore; ^3^ National Heart Research Institute Singapore National Heart Centre Singapore Singapore; ^4^ Institute of Biochemistry Medical School Justus‐Liebig University Giessen Germany; ^5^ Tecnologico de Monterrey Centro de Biotecnologia‐FEMSA Monterrey Mexico; ^6^ Institute of Fundamental Medicine and Biology Kazan (Volga Region) Federal University Kazan Russian Federation; ^7^ Vall d'Hebron Institut de Recerca University Hospital Vall d'Hebron‐Universitat Autònoma Barcelona Spain; ^8^ Centro de Investigación Biomédica en Red‐CV CIBER‐CV Spain; ^9^ Institute for Pathophysiology West German Heart and Vascular Center University of Essen. Medical School Essen Germany; ^10^ CarMeN Laboratory Hôpital Louis Pradel Hospices Civils de Lyon Université de Lyon and Explorations Fonctionnelles Cardiovasculaires INSERM U1060 Lyon France

**Keywords:** cyclosporine A, ischaemia, mitochondria, myocardial infarction, reperfusion

## Abstract

Pre‐clinical studies have indicated that mitoprotective drugs may add cardioprotection beyond rapid revascularization, antiplatelet therapy and risk modification. We review the clinical efficacy of mitoprotective drugs that have progressed to clinical testing comprising cyclosporine A, KAI‐9803, MTP131 and TRO 40303. Whereas cyclosporine may reduce infarct size in patients undergoing primary angioplasty as evaluated by release of myocardial ischaemic biomarkers and infarct size imaging, the other drugs were not capable of demonstrating this effect in the clinical setting. The absent effect leaves the role of the mitochondrial permeability transition pore for reperfusion injury in humans unanswered and indicates that targeting one single mechanism to provide mitoprotection may not be efficient. Moreover, the lack of effect may relate to favourable outcome with current optimal therapy, but conditions such as age, sex, diabetes, dyslipidaemia and concurrent medications may also alter mitochondrial function. However, as long as the molecular structure of the pore remains unknown and specific inhibitors of its opening are lacking, the mitochondrial permeability transition pore remains a target for alleviation of reperfusion injury. Nevertheless, taking conditions such as ageing, sex, comorbidities and co‐medication into account may be of paramount importance during the design of pre‐clinical and clinical studies testing mitoprotective drugs.

## 1 INTRODUCTION

Modern reperfusion therapy has improved outcome for patients with ST‐elevation myocardial infarction (STEMI) tremendously.^1^ Over the past 5 years, however, mortality reduction has levelled out^1^ and the decline in the incidence of post‐MI heart failure is modest.^2^ So, there still may be a need to reduce infarct size to further improve outcome.

Because infarct size depends on ischaemia time, the most important way to diminish it and improve outcome remains a reduction in the ischaemic time by reducing any delay and insuring rapid revascularization in STEMI patients. Beyond this focus, a major target may be an attempt to reduce infarct size by addressing the reperfusion injury that occurs, when injuring mechanisms are activated upon opening of the coronary artery.^3^, ^4^

Mitochondria in the heart are crucial for the generation of adenosine triphosphate (ATP) necessary to sustain contractile function and for the dynamic adjustment of the cardiomyocytes' metabolic demand and ionic homeostasis. Hence, the organelle is considered an important target for cardioprotection of the myocardium exposed to an acute ischaemia‐reperfusion injury. In particular, acute opening of the mitochondrial permeability transition pore (MPTP) has been involved in ischaemia‐reperfusion injury^5^, ^6^ because of its disruptive role in mitochondrial respiratory coupling and ATP production.^7^, ^8^ Experimental studies indicate that pharmacological approaches aimed at preventing MPTP have cardioprotective effects in the context of myocardial ischaemia reperfusion.^9^, ^10^ However, translation of this concept into the clinic has been disappointing,^11^, ^12^, ^13^, ^14^, ^15^, ^16^, ^17^ suggesting that targeting a single intracellular molecule, such as the MPTP or dynamin‐related protein 1 (Drp1),^18^ may not be sufficient to create cardioprotection.^19^, ^20^ It emphasizes that more mechanistic insight about the mode of action of cardioprotective modalities is needed. Lack of efficacy might also reflect that clinical outcome in STEMI patients undergoing primary percutaneous coronary intervention (PCI) is excellent by modern reperfusion therapy, such that ischaemia reperfusion as a target for protection has diminished. Median infarct size with current reperfusion therapy is small—in the order of magnitude of 7% and in anterior infarcts 16% of the left ventricle.^21^ Infarct sizes up to 17% rarely translate into clinical symptoms manifesting as cardiac death and hospitalization for heart failure,^22^ which are the most appropriate clinical end‐points for evaluating the efficacy of cardioprotective pharmacological agents.^23^

The aims of the present review were to provide an overview of the pharmacological agents that have advanced to clinical testing and to identify obstacles for a clinical benefit in order to clarify whether pharmacological mitoprotection is a useful way to pursue for improving outcome in STEMI patients undergoing reperfusion therapy.

## 2 PATHOPHYSIOLOGICAL BACKGROUND FOR INTERVENTION AGAINST MITOCHONDRIAL DYSFUNCTION IN ISCHAEMIA‐REPERFUSION INJURY

In a clinical context, mitochondrial dysfunction has been reported in cardiac diseases including ischaemia‐reperfusion injury as well as in comorbidities associated with ischaemic cardiomyopathies such as diabetes or obesity. Under aerobic conditions, mitochondria are indispensable for cell function and viability primarily through ATP production, regulation of cellular redox potential and control of apoptosis. Because of their close functional and anatomical association with the sarcoplasmic reticulum, mitochondria play an active role in calcium uptake, which in turn is a critical regulator of the Krebs cycle and therefore of mitochondrial respiration and NAPDH‐dependent antioxidant regeneration.^24^, ^25^, ^26^ Mitochondrial calcium uptake is driven by the mitochondrial calcium uniporter, whose low affinity is counteracted by the high calcium concentration present at the sarcoplasmic reticulum–mitochondria interface.^27^

Mitochondrial dysfunction can be of various origins and may alter cell homeostasis or viability through several mechanisms including reduction in ATP production, enhanced oxidative stress and release of pro‐apoptotic molecules. Following prolonged ischaemia reperfusion, the accumulation of calcium within the mitochondrial matrix and increased reactive oxygen species (ROS) production favour the opening of the MPTP. The opening of this non‐selective mega‐channel dissipates the mitochondrial electrochemical gradient necessary for ATP production and precipitates energy exhaustion and mitochondrial matrix swelling. Rupture of the outer mitochondrial membrane favours the release of pro‐apoptotic factors. During ischaemia, MPTP remains inhibited because of the acidic pH, but upon restoration of blood flow, rapid normalization of pH in the presence of calcium overload and excessive ROS triggers MPTP and exacerbates reperfusion‐induced cell injury.^5^ Various proteins have been reported to contribute in the formation and function of MPTP, including adenine nucleotide translocase (ANT), voltage‐dependent anion channel (VDAC) and the phosphate carrier, whereas others, including cyclophilin D (CypD), appear as key regulators. Its molecular identity remains, however, not fully elucidated. Bernardi et al have proposed that mitochondrial ATP synthase could be the true molecular entity of the MPTP, although this hypothesis remains controversial.^28^, ^29^

## 3 MECHANISMS UNDERLYING ATTENUATION OF MITOCHONDRIAL DYSFUNCTION IN ISCHAEMIA‐REPERFUSION INJURY BY PHARMACOLOGICAL AND MECHANICAL CONDITIONING APPROACHES

Based on the absence of a definite molecular structure of the MPTP and the existence of multiple modulators of its mechanics, a precise therapeutic strategy to prevent MPTP opening is difficult to establish. Pharmacological or non‐pharmacological (eg conditioning) interventions either aim at a particular target site of a putative component or modulator of the MPTP or try to prevent the conditions of its opening (eg calcium accumulation into the matrix) or to attenuate its consequences (eg altered oxidative phosphorylation). Based on encouraging in vitro and in vivo results, the ability of some pharmacological agents supposed to prevent MPTP opening has recently been tested in humans. Studies in isolated cardiac mitochondria from patients undergoing coronary artery bypass surgery had suggested improved mitochondrial function along with reduced troponin release with remote ischaemic pre‐conditioning.^30^

### 3.1 Cyclosporine A

**Figure 1:**
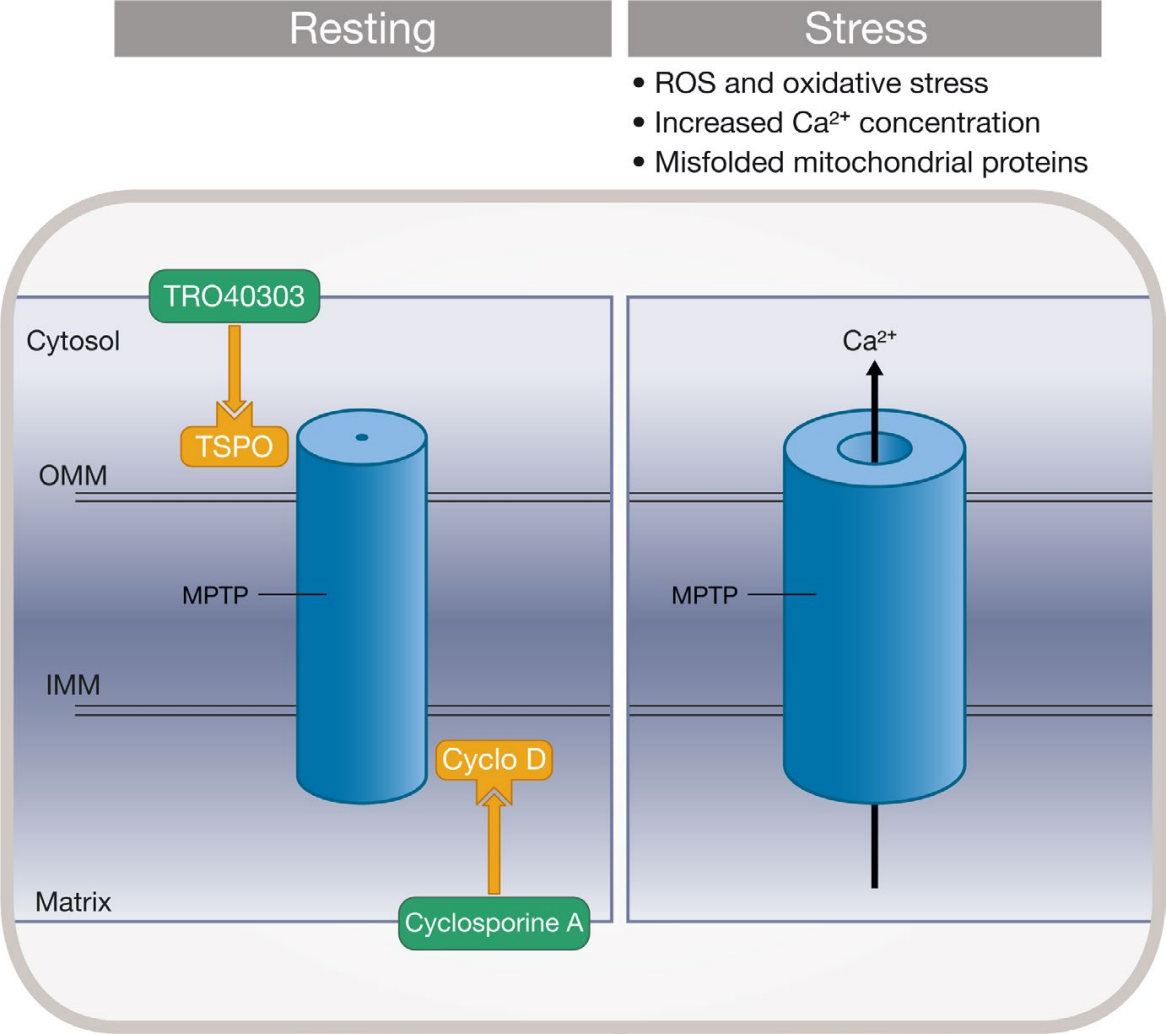
Cyclosporine A and TRO40303 inhibit opening of mitochondrial permeability transition pores (MPTP). Proteins implicated in MPTP formation include the matrix cyclophilin D (CyD), the inner membrane (IMM) and the outer mitochondrial membrane (OMM). Additional proteins such as the translocator protein 18 kDa (TSPO), located in the OMM, interact with proteins implicated in MPTP formation. Under pathophysiological conditions, such as high Ca^2+^ concentration and increased oxidative stress, the complex forms an open pore between the inner and outer membranes that ultimately result in mitochondrial swelling, mitochondrial Ca^2+^ efflux and the release of apoptogenic proteins. Cyclosporine A targets matrix CyD, where Ca^2+^ overload triggers MPTP opening. TRO40303 binds to TSPO in the outer membrane

Apart from the immunosuppressive action related to its binding to the cytosolic calcineurin and subsequent inhibition of the transcription factor nuclear factor of activated T cells (NFAT), cyclosporine A (CsA) inhibits MPTP opening following binding to the mitochondrial matrix chaperone CypD (Figure 1). CypD, which is known to bind to the oligomycin sensitivity conferral protein (OSCP), acts on the MPTP by facilitating the removal of the F_1_ domain from the c subunit in a CsA‐sensitive manner during pore opening.^31^ The concept of a putative cardioprotection by CsA was also based on the observation that CypD‐deficient mice develop significantly smaller infarcts after a prolonged ischaemic insult.^32^, ^33^ Hausenloy et al first reported that administration of CsA at the time of reperfusion could reduce infarct size in the isolated rat heart model.^34^ In vivo administration of NIM811, a non‐immunosuppressive CsA derivative, was able to inhibit MPTP opening in mitochondria isolated from reperfused rabbit myocardium and limit infarct size when administered at the time of reperfusion.^35^ CsA also reduced infarct size in anaesthetized pigs with 90‐minutes regional ischaemia, when given intravenously just before reperfusion.^36^ However, CsA failed to induce robust cardioprotection in other studies with efficacy dependency on the experimental conditions, such as duration of ischaemic period.^37^, ^38^ Also, CsA administered during reperfusion fails to restore cardioprotection in pre‐diabetic Zucker obese rats in vivo.^39^ Overall, CsA variably and inconsistently seems to reduce infarct size across species in experimental models of reperfused myocardial infarction.^40^

### 3.2 KAI‐9803

Although contentious,^41^ the protein kinase C (PKC) family of isoenzymes has been involved in pre‐conditioning protection against ischaemia‐reperfusion injury.^42^ The KAI‐9803 peptide (delcasertib) inhibits δ‐PKC activity and prevents translocation of δ‐PKC to the mitochondria during prolonged ischaemia reperfusion. Whereas administration of KAI‐9803 may preserve mitochondrial function, it has no direct action on MPTP opening, but would rather prevent apoptosis by limiting the accumulation and dephosphorylation of the pro‐apoptotic Bcl‐2–associated death promoter.^43^ In the in vivo pig model, intracoronary administration of KAI‐9803 immediately prior to reperfusion reduced infarct size and improved contractile function recovery.^44^

### 3.3 TRO 40303

TRO 40303 was initially presented as an inhibitor of MPTP opening.^10^ In vitro experiments suggested that this compound might reduce oxidative stress and subsequently prevent opening of the MPTP. Experimental evidence suggests that TRO40303 acts through its binding to the translocator protein (TSPO) located in the outer mitochondrial membrane (Figure 1). Importantly, Sileikyte et al demonstrated using calcium retention capacity measurements in isolated mitochondria that TRO40303 has no direct effect on the MPTP.^45^ However, it is important to emphasize that no targets and mechanisms for TRO40303 have been defined. Intravenous administration of 2.5 mg/kg of TRO40303 immediately prior to reperfusion reduced infarct size by 38% in the in vivo rat model of ischaemia reperfusion.^10^ However, in saline‐perfused rat hearts and in anaesthetized pigs, TRO40303 did not reduce infarct size when given at reperfusion.^46^

### 3.4 MTP131

MTP‐131 (Bendavia) may reduce infarct size, when administered at the time of reflow in various animal models. In isolated cardiac mitochondria, Zhao et al suggested that MTP‐131 was able to limit ROS production.^47^ Alternatively, an enhanced ROS scavenger capacity of MTP‐131 during ischaemia reperfusion has been discussed, but no specific target or mechanisms have been defined. In a rat model of acute kidney injury, MTP‐131 binds to cardiolipin, prevents its peroxidation by cytochrome c, thereby protecting mitochondrial cristae membranes during renal ischaemia reperfusion.^48^ Conflicting results regarding infarct size reduction have been reported suggesting a not so clear putative cardioprotection potential.^49^

## 4 CLINICAL STUDIES OF DRUGS TARGETING MITOCHONDRIAL FUNCTION AS AN ADJUNCT TO REPERFUSION IN ST‐SEGMENT ELEVATION MYOCARDIAL INFARCTION

An overview of clinical studies of mitoprotective drugs is given in the Table 1.

**Table 1:** Clinical trials investigating pharmacological agents for the treatment of ischaemia‐reperfusion injury in patients with ST‐elevation myocardial infarction undergoing primary percutaneous coronary intervention

Author	Year	Study design and number of patients	Treatment and follow‐up	Results
Piot et al^50^	2008	Multi‐centre, single‐blinded RCT (58 patients with STEMI and TIMI flow grade 0 undergoing primary PCI)	CsA (iv bolus 2.5 mg/kg, prior to PCI), infarct size quantification by CK and TnI release and CMR at 5 d post‐MI in a subgroup of 27 patients	44% reduction in CK (*P* = .04) 13% reduction in TnI (*P* = .15) 20% infarct size reduction by CMR (*P* = .04)
Mewton et al^51^	2010	Multi‐centre, single‐blinded RCT (28 patients with STEMI and TIMI flow grade 0 undergoing primary PCI)	CsA (iv bolus 2.5 mg/kg, prior to PCI), infarct size quantification and remodelling by CMR at 6 mo post‐MI	24% infarct size reduction (*P* = .04)
Ghaffari et al^11^	2013	Double‐blinded RCT (101 patients with anterior STEMI undergoing thrombolytic therapy)	CsA (iv bolus 2.5 mg/kg immediately before thrombolysis), infarct size evaluation by peak CK‐MB and TnT release, ST‐segment resolution and 6‐month clinical follow‐up	No significant effect
Cung et al^12^	2015	Multi‐centre, double‐blind, RCT (791 patients with anterior STEMI and TIMI flow grade 0 undergoing primary PCI)	CsA (iv bolus 2.5 mg/kg, prior to PCI), follow‐up at 12 mo by a composite end‐point (all‐cause mortality, worsening of heart failure during initial hospitalization, rehospitalization for heart failure or adverse left ventricular remodelling)	No significant effect
Ottani et al^13^	2016	Multi‐centre, open‐label RCT (410 patients with large STEMI and TIMI flow grades 0 to 1 undergoing primary PCI)	CsA (iv bolus 2.5 mg/kg, prior to PCI), end‐points ≥70% ST‐segment resolution 60 min after TIMI flow grade 3, TnT release and clinical outcome at 6 mo	No significant effect
Bates et al^15^	2008	Dose‐escalation safety study (154 patients with anterior MI	KAI‐9803 (delcasertib) 0.05 mg; 0.5 mg; 1.25 mg; 5.0 mg vs placebo i.c.), primary end‐point serious adverse event, but CK‐MB AUC, ST‐recovery AUC and infarct size by ^99^M‐technetium sestamibi also monitored	No safety and tolerability concerns Non‐significant reductions in CK‐MB release, improvements of ST recovery and (99m)technetium sestamibi infarct size
Linkoff et al^16^	2014	Multi‐centre, double‐blind RCT (1010 patient with anterior STEMI and 166 with inferior STEMI (an exploratory cohort) undergoing primary PCI)	KAI‐9803 (delcasertib) (50, 150 or 450 mg/h) by iv infusion prior to PCI and continued for approximately 2.5 h, infarct size quantification by CK (AUC).	No significant effect
Atar et al^14^	2015	Double‐blind, randomized trial (163 patients large STEMI and TIMI flow grades 0 to 1 undergoing primary PCI)	TRO40303 (iv bolus 6 mg/kg prior to PCI), infarct size quantification by CK, TnT and CMR	No significant effect. A larger number of adjudicated safety events in the TRO40303 group
Gibson et al^17^	2016	Multi‐centre, double‐blind phase 2A safety and efficacy RCT (118 first‐time anterior STEMI patients primary PCI)	MTP‐131 vs. placebo infused at a rate of 0.05 mg/kg/h for 1 h, infarct size by CK‐MB AUC, CMR and clinical outcome at 6 mo	No significant effect

Abbreviations: AUC, area under the curve; CK, creatine kinase; CK‐MB, creatine kinase myocardial band; CsA, cyclosporine A; PCI, percutaneous coronary intervention; RCT, randomized clinical trial; STEMI, ST‐elevation myocardial infarction; TIMI, thrombolysis in myocardial infarction; TnT, troponin T.

### 4.1 Cyclosporine A

The first seminal clinical proof of concept by Piot et al demonstrated that CsA 2.5 mg/kg iv <10 minutes before primary PCI with direct stenting yielded a 40% reduction in creatine kinase (CK) release over 72 hours in 58 patients with reperfused STEMI.^50^ The reduction on infarct size persisted at 6 months and was associated with less detrimental remodelling.^51^ In a subgroup of 27 patients, the absolute mass of the area of hyperenhancement on cardiac magnetic resonance imaging (MRI) was significantly reduced in the CsA group corresponding to a reduction in infarct size from 18% to 15% of the left ventricle.^50^ In the subsequent CIRCUS phase III trial, no benefit in clinical outcome (composite of all‐cause mortality, worsening of heart failure during the initial hospitalization, hospitalization for heart failure and adverse left ventricular [LV] remodelling) was demonstrated within 1 year after reperfused acute anterior STEMI by 2.5 mg/kg CsA intravenously before primary PCI in 971 patients.^12^ Because the study demonstrated a significant interaction between Killip class and treatment effect (*P* = .009), suggesting a beneficial effect of CsA in the subgroup of patients with acute myocardial infarction (AMI) complicated by heart failure, the authors recently presented a post hoc subgroup analysis of 97 patients with large anterior MI and Killip class ≥2. Although the composite end‐point at 1 year occurred less frequently in the CsA arm than in the control arm (35% vs 58%, *P* = .02), CsA was not associated with improved clinical outcome after adjustment for baseline characteristics (adjusted HR: 0.68 [0.3‐1.4]).^52^ Of note, the initial proof‐of‐concept studies and the CIRCUS trial used different solvents for CsA, and the solvent in the CIRCUS trial may have induced protection per se and thus have obscured differences between CsA and placebo.^53^

An absent effect of CsA was subsequently confirmed in the CYCLE (CYCLosporinE A in Reperfused Acute Myocardial Infarction) trial.^13^ A single intravenous CsA bolus (2.5 mg/kg) before primary PCI had no effect on ST‐segment resolution or high‐sensitive cardiac troponin T, and it did not improve clinical outcome or LV remodelling up to 6 months.^13^ In patients undergoing reperfusion therapy with thrombolysis, CsA did not have any effect on troponin T and CK myocardial band (CK‐MB) release or percentage ST resolution at 90 minutes after treatment.^11^

A more recent meta‐analysis did not demonstrate any significant differences between the CsA and placebo in terms of all‐cause death (OR: 1.21, 95% CI: 0.78‐1.87) and cardiovascular death (OR: 1.05, 95% CI: 0.66‐2.49).^54^

Whereas the lack of translation may reflect that improvement in treatment and outcome has diminished ischaemia reperfusion as a target for protection, it was surprising in both studies that it was not possible to confirm the immediate reduction in biochemical myocardial necrosis marker release observed in the original proof‐of‐concept study by Piot et al.^50^ However, this finding is not unique in trials evaluating cardioprotection in humans. The CONDI‐1 study^21^ and the LIPSIA CONDITIONING trial^55^ also failed to show a clear reduction in infarct size with biochemical markers despite improved myocardial salvage by SPECT or cardiac MRI. Also, the RIC‐STEMI trial on remote ischaemic conditioning^56^ and the NHLBI‐sponsored trial on ischaemic post‐conditioning^57^ reported better clinical outcome in the absence of significant infarct size reduction. Biochemical markers may not be sufficiently accurate to evaluate reduction within the range of infarct sizes seen in humans with contemporary reperfusion therapy. Also, our focus on infarct size reduction to improve clinical outcome may have been too narrow.^58^ We must pay more attention to also reduce coronary microvascular obstruction.^59^, ^60^, ^61^ In fact, in the NHLBI‐sponsored trial on ischaemic post‐conditioning, coronary microvascular obstruction on MRI was reduced along with better clinical outcome.^57^ Pharmacological agents that specifically target microvascular obstruction would be of interest, administered alone or in association with infarct size reducing agents. The so far mostly disappointing data do not exclude that ischaemia‐reperfusion injury can be a target for modification. We must possibly address more targets to translate protection into a clinical benefit. As for mitochondria, we must also direct our attention not only to cardiomyocyte, but also to endothelial fibroblast, and smooth muscle cell mitochondria.^62^

Modern reperfusion therapy also includes treatment with platelet inhibiting P_2Y12_ antagonists, which possess inherent cardioprotective capacity^63^, ^64^ such that the efficacy of additional cardioprotection may be increasingly difficult to demonstrate.^65^ In the CIRCUS trial, more than 90% of the patients were treated with P_2Y12_ antagonists.^12^

During cardiac surgery under cardiopulmonary bypass with ischaemic cardioplegic arrest, 2.5 mg/kg CsA intravenously reduced cumulative troponin I release in patients undergoing aortic valve surgery.^66^ Similarly, CsA also reduced cumulative troponin T and CK‐MB release in patients with the longest cardiopulmonary bypass time of 120 (range: 85‐120 minutes) as compared with those with a shorter bypass time in the rage of 50‐85 minutes during aortocoronary bypass surgery.^67^ The same dose of CsA did not reduce release of cardiac troponin I or CK‐MB or incidence of arrhythmias by administration before thrombolysis with streptokinase in 101 patients with acute anterior STEMI.^11^

Noteworthy, there has been no evidence from the above‐mentioned trials that in these clinical conditions of STEMI, CsA had any detectable biological effect, thereby questioning our ability to deliver it in due time at the appropriate dose to the right molecular target. Although the magnitude of reperfusion injury may be too small for a modification that translates into a clinically important benefit, the emerging evidence that CsA may not have effect on infarct size leaves the role of the MPTP in reperfusion injury in humans unanswered because no MPTP inhibitors are available. As long as its molecular structure remains unknown and specific inhibitors of its opening are lacking, MPTP remains a candidate to alleviate reperfusion injury.

### 4.2 KAI‐9803

The selective inhibitor of delta‐protein kinase C (delta‐PKC), KAI‐9803, was first studied in a phase I dose‐escalation safety study of 154 patients with acute STEMI (DELTA‐MI trial).^15^ Doses were 0.05 mg, 0.5 mg, 1.25 mg and 5.0 mg vs placebo administrated in two divided doses via intracoronary injection before and after reestablishment of antegrade epicardial flow by primary PCI in patients with documented totally occluded vessels. The incidence of serious adverse events was not different in patients treated with KAI‐9803 vs placebo. Other safety end‐points, including changes in QT intervals and standard laboratory values after study drug administration, also did not differ between treatment groups. The study was not powered to demonstrate efficacy by biochemical myocardial necrosis marker release. However, it suggested evidence for drug activity by trends for consistent, non‐significant reductions in CK‐MB area under the curve and ST‐recovery area under the curve values across all dosages of KAI‐9803 compared with placebo. A similar trend was demonstrated for improvements in ^99^M‐technetium sestamibi infarct size with the active study drug at all doses of intracoronary KAI‐9803.^15^ However, the subsequent PROTECTION AMI international, multi‐centre, double‐blind trial, which also used a dose‐response approach (50, 150 or 450 mg/h vs placebo intravenously) as soon as possible after randomization, before the first contrast injection during PCI, and continued until all study drug had been administered (∼2.5 hours), in patients presenting within 6 hours and undergoing primary PCI for anterior (the primary analysis cohort, n = 1010 patients) or inferior (an exploratory cohort that was capped after inclusion of 166 patients) STEMI, demonstrated no reduction in circulating biochemical markers of myocardial injury measured by CK‐MB fraction area under the curve.^16^ Similarly, there were no treatment‐related differences in secondary end‐points of infarct size, electrocardiographic ST‐segment recovery measured as area under the curve or time to stable ST recovery, or LV ejection fraction at 3 months. Adjudicated clinical end‐points (death, heart failure or serious ventricular arrhythmias) also did not differ between the study groups. The doses used in the PROTECTION AMI trial had been calculated to provide steady‐state blood concentrations similar to or exceeding the levels that prevented reperfusion injury in animal models and to deliver cardiac tissue doses similar to those with intracoronary administration in the original DELTA‐MI trial. Circulating levels of KAI‐9803 were not measured in PROTECTION AMI. Even the highest dose of KAI‐9803 used in PROTECTION AMI may have been insufficient by intravenous administration, as the dose may not have secured an equivalent tissue concentration as the intracoronary administration used in the DELTA‐MI study. A lack of any side effect leaves the question unanswered whether higher doses may have been feasible and effective. Differences in the design of the DELTA‐MI study and the PROTECTION AMI trial studies may have been relevant, particularly because patients with totally occluded vessels were included in the DELTA‐MI study while approximately one‐third of patients in the PROTECTION AMI trial had spontaneous reperfusion prior to PCI. Finally, the favourable trends in the original DELTA‐MI may have been merely incidental in the small trial with multiple dosing arms. Delta‐PKC may not be of sufficient significance as a target for prevention of reperfusion injury despite some experimental mechanistic evidence, which remains somewhat controversial.^41^

### 4.3 TRO 40303

The single clinical study of TRO 40303 was the multi‐centre proof‐of‐concept MITOCARE study,^14^ in 163 patients with STEMI, totally occluded culprit vessels and large area at risk. The patients received TRO40303 (6 mg/kg), or placebo intravenously as a slow bolus (35 mL/min; given over approximately 1 minute) before guide wire passage, 15 minutes prior to balloon inflation and stenting. The primary end‐point was infarct size expressed as area under the curve for CK and for troponin I over three days. The result was neutral. In a subgroup of 93 patients, there were no significant differences in CMR‐assessed myocardial salvage index (1—infarct size/myocardium at risk) (mean 52% vs 58% with placebo, *P* = .1), mean CMR‐assessed infarct size (21.9 g vs 20.0 g, corresponding to 17% vs 15% of LV mass) or LVEF (46% vs 48%), or in the mean 30‐day echocardiographic LVEF (51.5% vs 52.2%) between TRO40303 and placebo. One of the reasons for the neutral result might be that the experimental background to justify a proceeding to a proof‐of‐concept clinical study was not sufficiently solid in relation to the choice of dose. Rat studies have shown a dose‐response–dependent infarct reduction in TRO40303.^10^, ^68^ A closer look on the data underlying this conclusion demonstrates uncertainties. The area at risk varied unexpectedly, and infarct sizes were modest in the model. Importantly, infarct size related to area at risk did not decrease with the highest TRO40303 dose but increased significantly in the vehicle group—not because of a change in infarct size but because of a change in area at risk. As a consequence, the relative reduction in infarct size compared with vehicle did not seem convincing. Concentrations in rats were lower than those achieved in humans. In saline‐perfused rat hearts and in anaesthetized pigs, TRO40303 did not reduce infarct size when given at reperfusion.^46^ Circulating levels of the drug were not measured in the MITOCARE study, and adjudicated side effects were frequent in the treatment group perhaps indicating that the dose might not have been optimal. However, the lack of effect and demonstration of side effects have prevented further testing of TRO40303.

### 4.4 MTP131

The EMBRACE STEMI^17^ was undertaken to study whether the clinical efficacy of the cell‐permeable peptide that preserves the integrity of cardiolipin, MTP‐131, enhances mitochondrial energetics and improves myocyte survival during reperfusion. The study included 118 patients with first‐time anterior STEMI because of a proximal or middle LAD lesion, who underwent successful PCI. Administration of MTP‐131 was safe and well‐tolerated. Treatment with MTP‐131 was not associated with a decrease in myocardial infarct size as assessed by area under the curve of CK‐MB within 0‐72 hours. No effects were observed on secondary end‐points including troponin I, volume of infarcted myocardium by cardiac MRI, measures of myocardial structure and function TIMI flow grade, ST‐segment elevation resolution or clinical outcome.^17^ As such, MTP‐131 adds to the number of potential mitoprotective pharmacological compounds that do not translate into a clinical benefit in patients with STEMI undergoing reperfusion therapy.

## 5 CHALLENGES IN TRANSLATING MITOPROTECTION INTO THE CLINICAL SETTING

The lack of clinical efficacy by agents that target mitochondria to confer cardioprotection might be understood in terms of redundancy of mitochondrial pathways, insufficient pathophysiological knowledge on the role of mitochondria in cell death/survival pathways, limited information about their cellular origin (cardiomyocyte vs coronary vascular), their structures and functions, and the complex interplay of the conditions closely related to cardiovascular disease that affects mitochondria, such as ageing, comorbidities and co‐medication.^69^, ^70^, ^71^

### 5.1 Ageing

As lifespan has extended, age‐related conditions accumulate in elder people. Some of these conditions disrupt mitochondrial functions. Ageing by itself induces a progressive impairment of mitochondrial respiratory efficiency as well as changes in mitochondrial morphology and mitochondrial pool^72^ that collectively account for the exercise intolerance seen in elderly patients.^73^ The aerobic capacity of the human myocardium is depressed in elderly people (>75 years) because of an excessive mitochondrial calcium accumulation that eventually leads to less number of respiring mitochondria.^74^ This mechanism may facilitate the transition from a healthy to a failing cardiomyocyte and could underlie the reduced tolerance to ischaemic damage observed in elderly patients^74^ and aged animals.^75^ The interfibrillar mitochondria are particularly sensitive to the effects of ageing, whereas subsarcolemmal mitochondria remain rather preserved.^76^, ^77^ During ageing, mitochondria also experience structural changes, including reduced inner membrane surface and disarrangement of cristae.^78^ Furthermore, impairment of the electron transfer chain complexes III and IV may be the cause of the disorganized ‘respirosomes’ observed in the aged heart.^79^ Other functional alterations of mitochondria of aged cardiac cells are excessive ROS production that favours mitochondrial DNA (mtDNA) damage,^80^ diminished mitophagy^81^ and an increased susceptibility to undergo MPTP opening upon reperfusion.^75^ Telomerase is not only present in the nucleus, but also in mitochondria, and telomerase abundance typically decreases with ageing and pre‐disposes to myocardial ischaemia‐reperfusion injury.^82^ Telomerase‐deficient rat hearts have increased infarct size.^83^ As a result of the wide range of altered mitochondrial mechanisms because of the natural process of ageing, pharmacological agents aimed at promoting mitoprotection might fail.

### 5.2 Sex differences

Experimental observations have confirmed the results of epidemiological studies investigating sex‐specific differences in cardiac tolerance to ischaemia.^84^

Female gender appears to favourably influence cardiac remodelling after ischaemia‐reperfusion injury. Detailed mechanisms of sex‐related differences remain unknown and may involve genomic and non‐genomic effects of sex steroid hormones, particularly the oestrogens, which have been the most extensively studied, but also by influences during the early‐phases of ontogenetic development. Experimental studies of the mitochondrial proteome have identified a number of mitochondrial proteins that have male/female differences in post‐translational modification.^85^ Specifically, males have increased phosphorylation of the pyruvate dehydrogenase (PDH)‐E1α subunit, whereas females have increased phosphorylation of mitochondrial aldehyde dehydrogenase‐2 (ALDH2), an enzyme involved in post‐ischaemic reperfusion and remote conditioning pathways,^86^, ^87^ and the E2 subunit of α‐ketoglutarate dehydrogenase.^85^ Therefore, females exhibited reduced ROS generation on reoxygenation. Similar to CsA, oestrogen may contribute to maintaining mitochondrial function during ischaemia reperfusion by stabilizing the mitochondrial membrane potential and inhibiting MPTP opening.^88^ Also, the protection during conditions of increased contractility seems to involve an increase in nitric oxide signalling that leads to S‐nitrosylation of the L‐type calcium channel. The nitrosylation reduces calcium loading during ischaemia and early reperfusion, and hence moderates ischaemia‐reperfusion injury.^89^ Hence, sex‐related differences in cardiac sensitivity to ischaemia‐reperfusion injury may influence mitoprotective strategies in patients with acute coronary syndrome.

### 5.3 Comorbidity and risk factors

Diabetes seems to attenuate the cardioprotective effects of pharmacological and ischaemic conditioning manoeuvers.^90^ This may partly be because of dysregulation of mitochondrial homeostasis, by impairing autophagy, overproduction of ROS, lipotoxicity and activation of calpain that has been linked to F‐ATPase proteolysis.^91^, ^92^ After an AMI, cardiomyocytes of type 2 diabetes mellitus rats present a significant down‐regulation of genes involved in mitochondrial fusion and autophagy when compared with non‐diabetic animals, manifesting as accumulation of incompletely degraded mitochondria and enhanced apoptosis of cardiomyocytes. These findings correlate with a severe form of heart failure and increased mortality among diabetic rats.^93^ Accordingly, cardiac cells of type 2 diabetic mice exhibit degenerated swollen mitochondria with disintegrated cristae and sparse autophagosomes, linked with the reduction in the phosphorylated state of the 5' adenosine monophosphate‐activated protein kinase (AMPK).^94^ In non‐diabetic animals, two‐week administration of resveratrol reverses heart remodelling after myocardial infarction via up‐regulated autophagy mediated by AMPK activation.^95^ Additionally, resveratrol improved LV diastolic and endothelial function in patients with a history of myocardial infarction, among whom nearly one‐third were diabetic.^96^ To what extent these last findings are generally applicable to diabetic patients remains to be proven. Loss of cardiomyocyte‐restricted insulin signalling decreases the mitochondrial capacity of fatty acid catabolism.^97^

Dyslipidaemia affects the myocardium directly, obscuring and preventing cardioprotection provided by ischaemic conditioning and pharmacological agents.^98^ This might be consequence of MPTP opening secondary to decreased levels of antioxidant enzyme expression^99^ or to reduced glycogen synthase kinase (GSK)‐3β phosphorylation.^100^ The MPTP opening inhibitor CsA cannot by its own offer cardioprotection in hypercholesterolaemic animals.^101^ Another target of mitoprotection is the mitochondrial potassium‐ATP (K_ATP_) channel located at the inner membrane. In hyperlipidaemic rats, neither cromakalim nor diazoxide, two K_ATP_ channel activators, maintain their cardioprotective effects in the context of ischaemia reperfusion.^102^

### 5.4 Co‐medication

At the same time as comorbidities may influence the efficacy of mitoprotective interventions, drugs used as treatments may alter mitochondrial processes by interference with the mitochondrial respiratory chain (eg uncoupling) or inhibition of important mitochondrial processes including oxidative phosphorylation, mitochondrial DNA replication and ADP/ATP translocation.

#### 5.4.1 Antidiabetic drugs

The antidiabetic compounds glibenclamide and other sulphonylureas promote MPTP opening, causing calcium efflux to the cytosol and resulting in swollen mitochondria.^103^ Conversely, metformin lowers mitochondrial oxidative stress in cultured human endothelial cells subjected to low glucose concentrations.^104^ Metformin may be a pharmacological modulator of MPTP opening,^105^ but it failed to improve LVEF at four months after STEMI in patients without diabetes undergoing primary PCI.^106^ Continuous delivery of metformin to a murine model of dilated cardiomyopathy increases mitophagy through AMPK phosphorylation and results in milder fibrosis and reduced LV hypertrophy.^107^ The selective sodium‐glucose cotransporter (SGLT2) inhibitor, canagliflozin, and the glucagon‐like peptide‐1 (GLP‐1) receptor antagonist, exenatide, attenuate myocardial infarction size.^108^, ^109^ Mechanisms may involve improvement of mitochondrial function through the GLP‐1 receptor/cAMP/PKA pathway.^109^ Hence, type 2 diabetes mellitus per se, along with its medication, could be major confounders in clinical trials aimed to prove mitoprotective agents.

#### 5.4.2 Lipid‐lowering drugs

Statins inhibit mitochondrial respiration, stimulate ROS production and may facilitate MPTP opening.^110^, ^111^ A proprotein convertase subtilisin/kexin 9 (PCSK9) inhibitor as well as high doses of atorvastatin lowers the ratio between phosphorylated and total Drp‐1, a key factor in mitochondrial fission, whereas only the PCSK9 inhibitor restores mitochondrial ROS levels in insulin‐resistant, dyslipidaemic rats.^112^ Expression of PCSK9 is increased in the viable reperfused myocardium and its pharmacological inhibition can reduce infarct size, possible through autophagy, in the in vivo mouse model of myocardial infarction.^113^ Paradoxically, although some statins such as pravastatin seem to facilitate MPTP opening, atorvastatin reduces infarct size in the isolated mouse‐perfused hearts by activating the Akt‐eNOS pathway,^114^ suggesting that MTPT modulation may vary between statins. Mitochondrial damage is not restricted to cardiomyocytes, but it is also encountered in other cell types. In pigs with metabolic syndrome, mitochondrial density is diminished in LV endothelial cells, and accordingly, endothelium‐dependent vasorelaxation of coronary arteries is compromised. Of note, regular exercise seems to exert mitoprotective effects even in dyslipidaemic conditions.^99^

#### 5.4.3 Chemotherapeutics

Widely prescribed chemotherapeutics display a plethora of adverse side effects mediated by mitochondria damage with cardiotoxicity as the most feared.^115^, ^116^ This is the case for the inhibitor of topoisomerase II, doxorubicin and other anthracyclines, which stand as the main cause of chemotherapy‐related cardiotoxicity.^117^ Doxorubicin diverts electrons from respiratory chain complex I and other dehydrogenases that induce ROS overproduction.^118^ Despite its intended effect is preventing DNA replication at the nucleus of cancer cells, doxorubicin also reduces mtDNA levels and thus mitochondrial biogenesis in all tissues, including the myocardium.^119^ Indirectly, doxorubicin triggers the irreversible accumulation of mtDNA adducts through ROS production in a cardioselective manner.^120^ The modes of action of mitoxantrone and doxorubicin present a high degree of similarity.^121^ Mitoxantrone hampers oxidative phosphorylation, whereas its associated ROS production is relatively limited and secondary to ATP depletion.^122^ Nevertheless, it mitoxantrone inhibits the mitochondrial calcium uniporter by a direct interaction.^123^ The tyrosine kinase inhibitors, trastuzumab, is an antibody directed against the human epidermal growth factor receptor 2 that has as an adverse side effect of the heightened risk of congestive heart failure.^124^ Trastuzumab cardiomyopathy could be because of the disruption of critical signalling that sustains cardiomyocyte survival,^125^ but mitochondrial dysfunction and ROS imbalance cannot be dismissed.^126^ Other anticancer drugs, including tamoxifen, flutamide, alkylating agents and taxanes, produce considerable alterations in essential mitochondrial functions. On the other hand, anti‐proliferative drugs such as rapamycin/sirolimus that are used in drug‐eluting stents after revascularization could owe their success partially to induced mitophagy and reduced apoptosis after ischaemia reperfusion, as observed in other tissues.^127^ Despite their modulatory effect on mitochondrial function, the interaction between chemotherapeutics and specific mitoprotective strategies in the clinical setting remains unknown.

#### 5.4.4 Non‐steroidal anti‐inflammatory drugs

Non‐steroidal anti‐inflammatory drugs (NSAIDs) such as naproxen, diclofenac and celecoxib have been associated with an increased risk of cardiovascular disease, predominantly coronary thrombosis, because of their variable affinity to the cyclooxygenase 1 and cyclooxygenase 2 (COX‐1 and COX‐2) enzymes that may alter thrombogenicity.^128^ Recent experimental studies on isolated rat heart mitochondria have demonstrated that NSAIDs may also increase ROS formation, mitochondria membrane collapse, mitochondria swelling, lipid peroxidation, and glutathione and ATP depletion,^129^ which may play important roles in developing cardiotoxicity. MPTP sealing agents and antioxidants may prevent mitochondrial toxicity.^129^ However, the clinical implications of these observations remain unknown.

## 6 PERSPECTIVE AND CONCLUSION

Despite pre‐clinical evidence for a cardioprotective effect, most firmly established for CsA, neither of the clinically tested mitoprotective drugs has demonstrated protective capability on clinical outcome beyond that provided by rapid revascularization alone. Whereas it may relate to favourable outcome with current optimal therapy, risk factors, comorbidity and concurrent medications may also alter mitochondrial function, sometimes in an irreversible way and often by multiple mechanisms. In consequence, targeting one single mechanism to provide mitoprotection may be ineffectual. However, as long as the molecular structure of the MPTP remains unknown and specific inhibitors of its opening are lacking, it remains a candidate to alleviate reperfusion injury. Nevertheless, taking conditions such as ageing, sex, comorbidities and co‐medication into account may be of paramount importance during the design of pre‐clinical and clinical studies testing mitoprotective drugs.

## CONFLICT OF INTEREST

None.

## AUTHOR CONTRIBUTIONS

All authors contributed equally to the manuscript.

## Data Availability

Data sharing is not applicable to this article as no new data were created or analysed in this study.
